# Supplementary material for: MOTS-c is an exercise-induced mitochondrial-encoded regulator of age-dependent physical decline and muscle homeostasis
Source: Nat Commun. 2021 Jan 20;12:470. doi: 10.1038/s41467-020-20790-0 (PMC7817689; doi:10.1038/s41467-020-20790-0)
Supplement: Supplementary file 1 — Supplementary Information [file 41467_2020_20790_MOESM1_ESM.pdf]

Supplementary Information for:

MOTS-c is an Exercise-Induced Mitochondrial-Encoded Regulator of Age-Dependent Physical Decline and Muscle Homeostasis

Authors: Joseph C. Reynolds[1], Rochelle W. Lai[1], Jonathan S.T. Woodhead[2,3], James H. Joly[4], Cameron J. Mitchell[5,6], David Cameron-Smith[5], Ryan Lu[1], Pinchas Cohen[1,7], Nicholas A. Graham[4,7], Bérénice A. Benayoun[1,7,8], Troy L. Merry[5,6], Changhan Lee[1,7,9]*.

[1] Leonard Davis School of Gerontology, University of Southern California, Los Angeles, CA 90089.

[2] Discipline of Nutrition, Faculty of Medical and Health Sciences, The University of Auckland, Auckland, New Zealand.

[3] Maurice Wilkins Centre for Molecular Biodiscovery, The University of Auckland, Auckland, New Zealand.

[4] USC Mork Family Department of Chemical Engineering and Materials Science, Los Angeles, CA 90089.

[5] Liggins Institute, The University of Auckland, Auckland, New Zealand.

[6] School of Kinesiology, University of British Colombia, Vancouver, BC, Canada V6T 1Z3.

[7] USC Norris Comprehensive Cancer Center, Los Angeles, CA 90089.

[8] USC Stem Cell Initiative, Los Angeles, CA 90089.

[9] Biomedical Science, Graduate School, Ajou University, Suwon 16499, Korea.

*corresponding author: changhan.lee@usc.edu

This file includes Supplementary Figures 1-18.

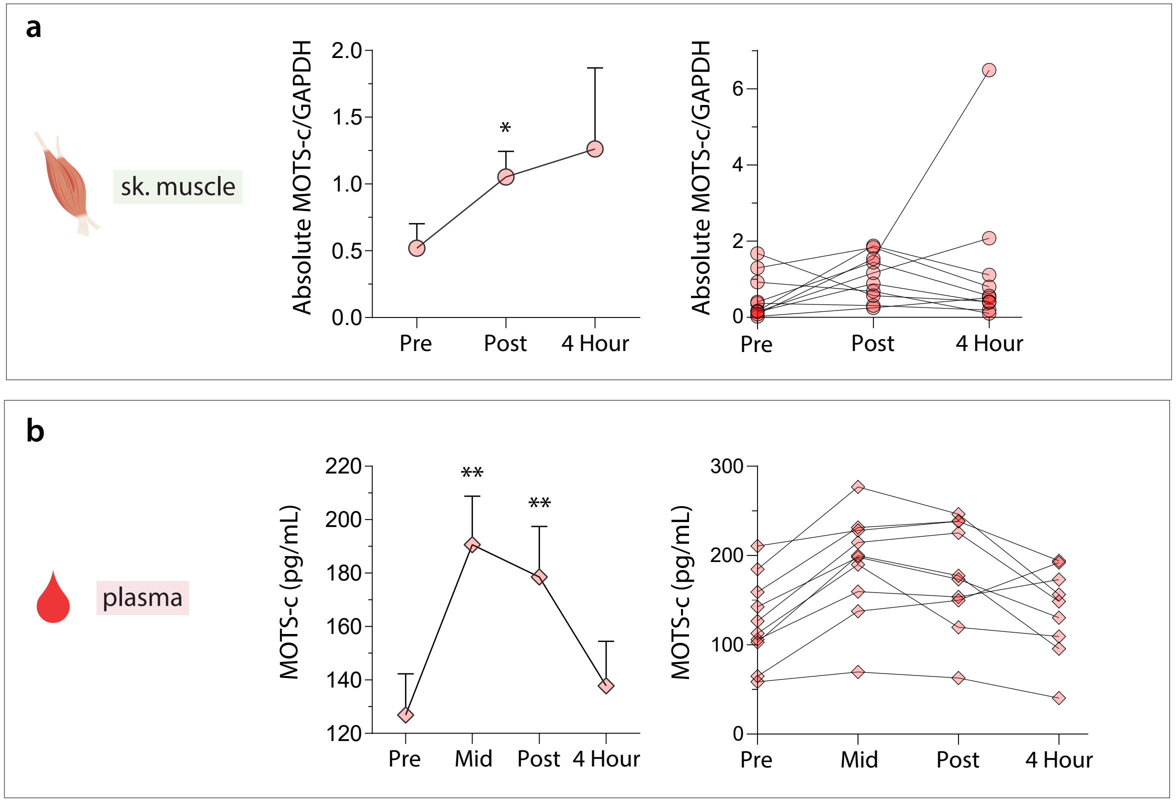

Supplementary Figure 1. Absolute MOTS-c levels in human muscle and plasma. Relative MOTS-c levels are shown in Fig. 1c-d. Here, the absolute quantification of MOTS-c levels measured by **a** Western blotting on human skeletal muscle collected pre-, post-exercise and 4-hours of resting, normalized to corresponding GAPDH levels (MOTS-c/GAPDH) (*P*=0.0098) and **b** ELISA on plasma from the same individuals collected pre-, mid-, post- exercise and 4-hours of resting (n=10) (*P*=0.0011 Mid-; *P*=0.0020 Post-exercise). Data are presented as both average values and individual datapoints. Statistics by Wilcoxon matched-pair signed rank test. *$P<0.005$ **$P<0.01$.

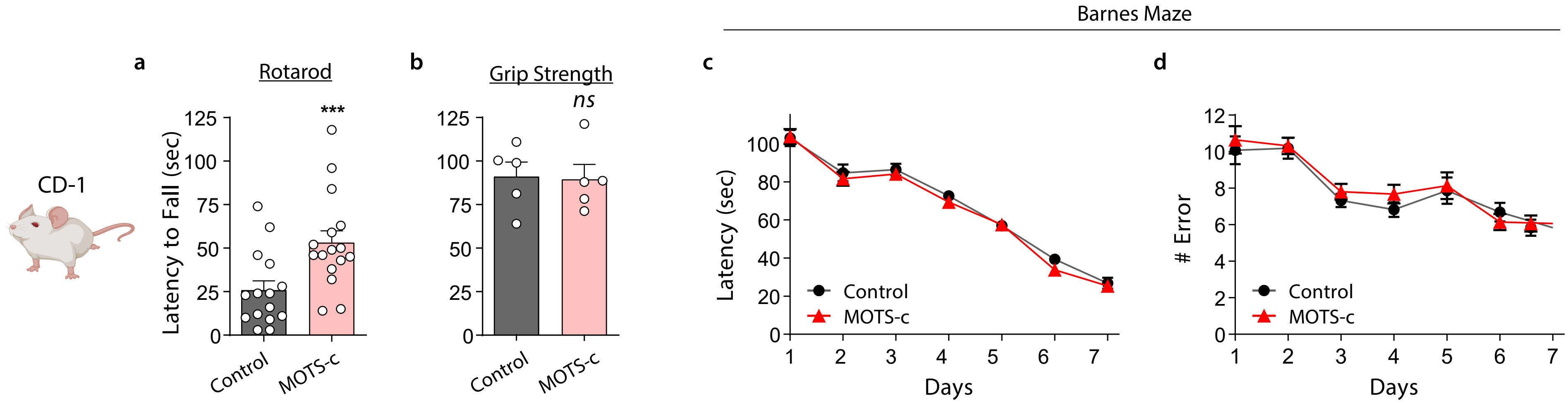

Supplementary Figure 2. Rotarod, grip strength, and Barnes Maze tests in MOTS-c treated old mice. a Summary of latancy time to fall on the Rotarod test (n=13 Control, n=16 MOTS-c) (*P*=0.0042). The speed of the rotations increased from a starting speed of 24 rpm by 1 rpm every 10 seconds. **b** Grip strength test (n=5) (*P*=0.7012). **c,d** Barnes Maze performance in control and MOTS-c treated 12-week old mice (n=15). **c** There was no change in the average time to find the escape box (latency) between control and MOTS-c treated mice. **d** There was no change in the number of errors made prior to discovering the escape box between groups. Errors were defined as nose-pokes or head deflections over false holes. Data expressed as mean +/- SEM of three 24-hour acquisition cycles. Two-sided Student's t-test. *$P<0.05$. **$P<0.01$, ***$P<0.0001$.

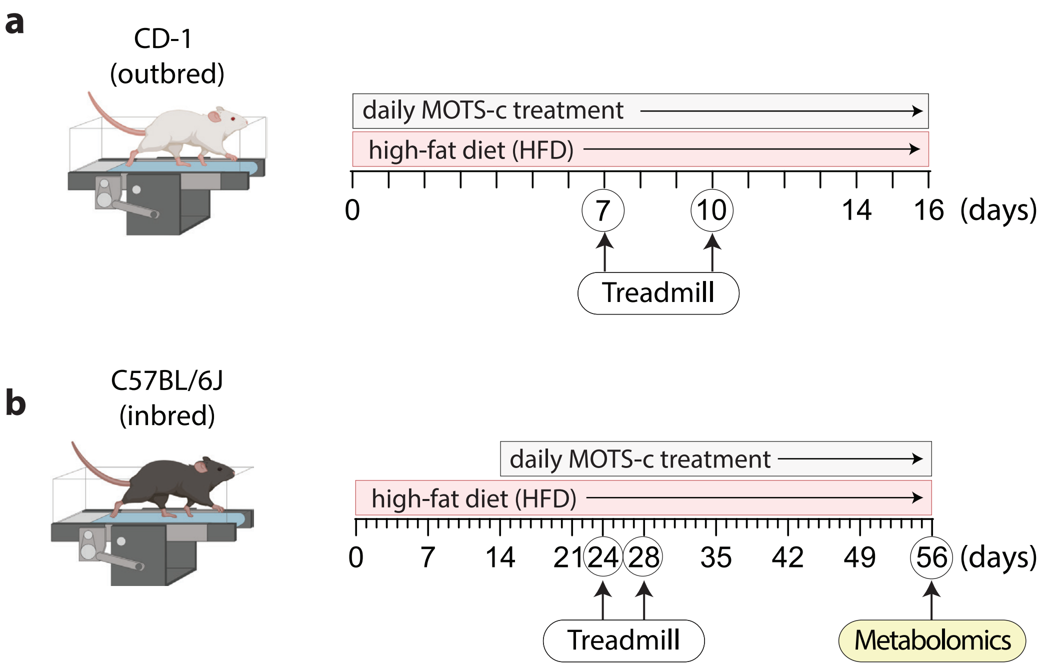

Supplementary Figure 3. Outline of HFD mouse experiments. Timeline of experiment for 12-week old male CD-1 (outbred) and C57BL/6J (inbred) mice fed a HFD or defined control diet. **a** CD-1 mice were fed a HFD and given daily intraperitoneal injections (IP) of MOTS-c (0, 5, or 15 mg/kg/day) from Day 0. Treadmill running tests were performed on Day 7 (Supplementary Fig. 5a) and Day 10 (Fig. 2a-d). Daily MOTS-c injections ceased at Day 16. **b** C57BL/6J mice were started on either a HFD or a defined control diet on Day 0 and continued uninterruptedly throughout the experiment. Daily MOTS-c treatment (15 mg/kg; IP) started on Day 14. Treadmill running tests were performed on Day 24 and Day 28 (10 days and 14 days after the start of MOTS-c treatment) (Fig. 2e-h; Supplementary Fig. 5b). Mice were treated daily until Day 56, at which time metabolomics was performed (Fig. 2i).

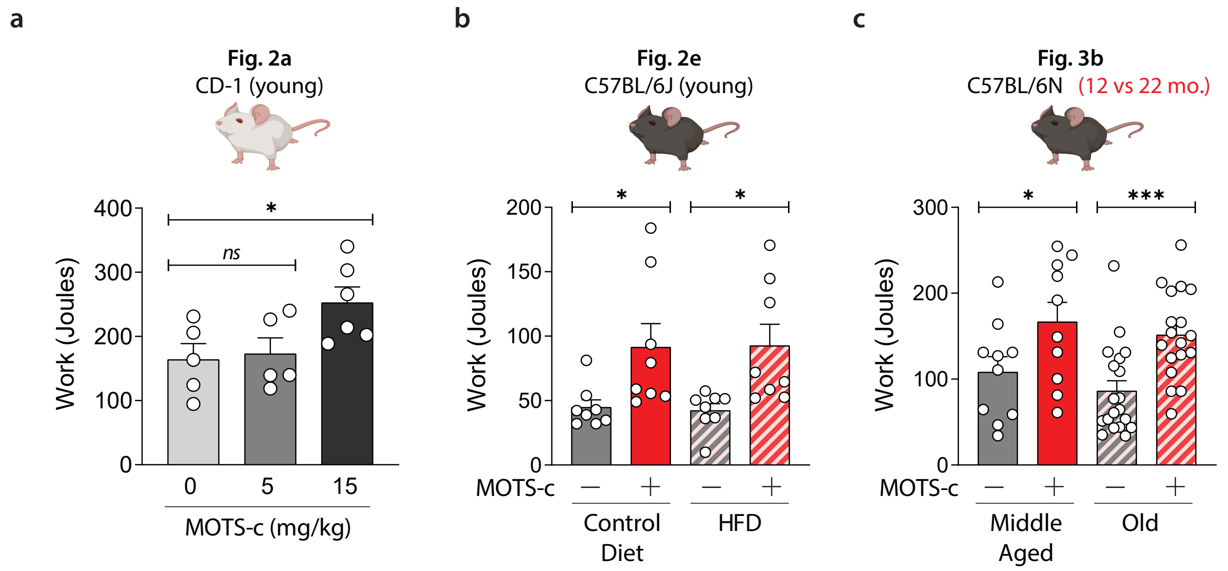

Supplementary Figure 4. The effect of MOTS-c on exercise work output. Based on the treadmill running studies in **a** young CD-1 (Fig. 2a) (n=5 0,5 mg/kg MOTS-c, n=6 15mg/kg MOTS-c) (*P*=0.0346), **b** C57BL/6J (Fig. 2e) (n=8) (*P*=0.0286 control diet; *P*=0.0122 HFD), and **c** middle-aged and old C57BL/6N mice (Fig. 3b) (n=10 Middle-Aged mice, n=18 Old MOTS-c and n=19 Old Control) (*P*=0.0479 Middle-Aged and *P*=0.0005 Old), total exercise work output was calculated taking into account body weight, acceleration due to gravity, and distance. Data expressed as mean +/- SEM. Two-sided Student'ds t-test **P*<0.05, *****P*<0.0001.

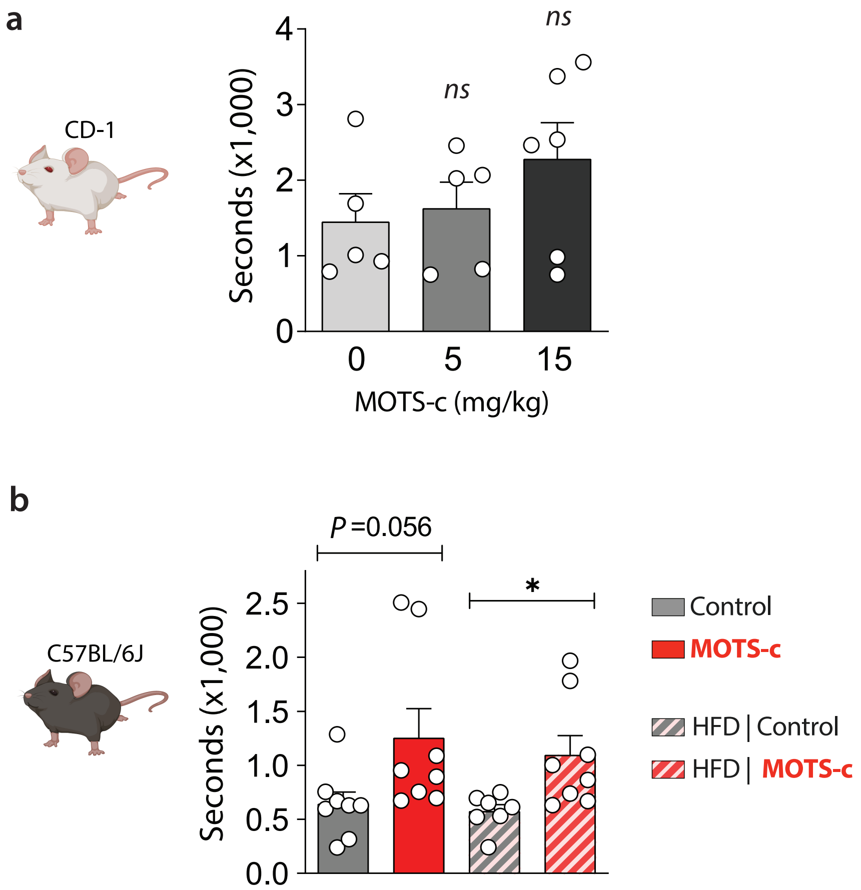

Supplementary Figure 5. Initial running time of MOTS-c treated young mice. a Running time of CD-1 mice following seven days of MOTS-c treatment (n=5 for Control and 5 mg/kg MOTS-c, n=6 for 15 mg/kg MOTS-c). MOTS-c (15mg/kg/day) treatment showed a trend towards enhanced running performance. **b** Running time of HFD-fed C57BL/6J mice following 10 days of MOTS-c treatment (n=8) (*P*=0.0229). Data expressed as mean +/- SEM. Two-sided Student's t-test. **P*<0.05

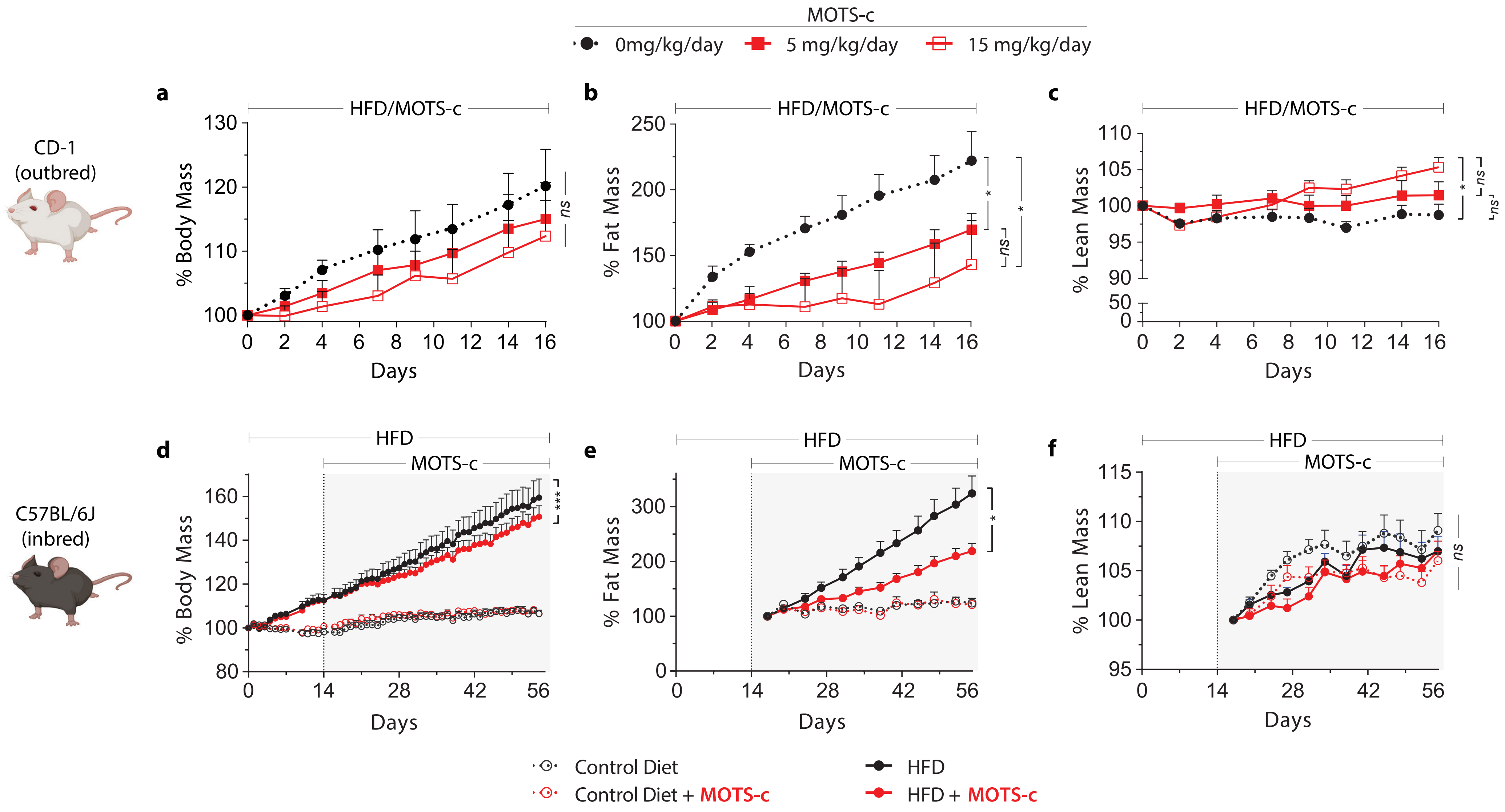

Supplementary Figure 6. Body composition analysis on MOTS-c treated young mice. Body composition was measured non-invasively using a time-domain NMR analyzer. **a-c** Young CD-1 mice were treated daily with MOTS-c (0, 5, or 15 mg/kg/day;IP) for 16 days (n=5 control and 5 mg/kg/day MOTS-c, n=6 for 15 mg/kg/day MOTS-c and percent **a** body weight, **b** fat mass (*P*=0.0320, 5 vs. 15; *P*=0.0251, 0 v 15), and **c** lean muscle mass (*P*=0.0105) were measured. **d-f** C57BL/6J mice either on a HFD or a defined Control Diet and treated daily with MOTS-c (15 mg/kg/day;IP) or saline control (n=8) and percent **d** body weight (*P*<1E-15), **e** fat mass (*P*=0.0119), and **f** lean muscle mass were measured. The dotted line at Day 14 represents the start of MOTS-c treatment. Data expressed at mean +/- SEM. Significance determined using two-wat ANOVA (repeated measures). **P*<0.05, ***P*<0.001, ****P*<0.0001.

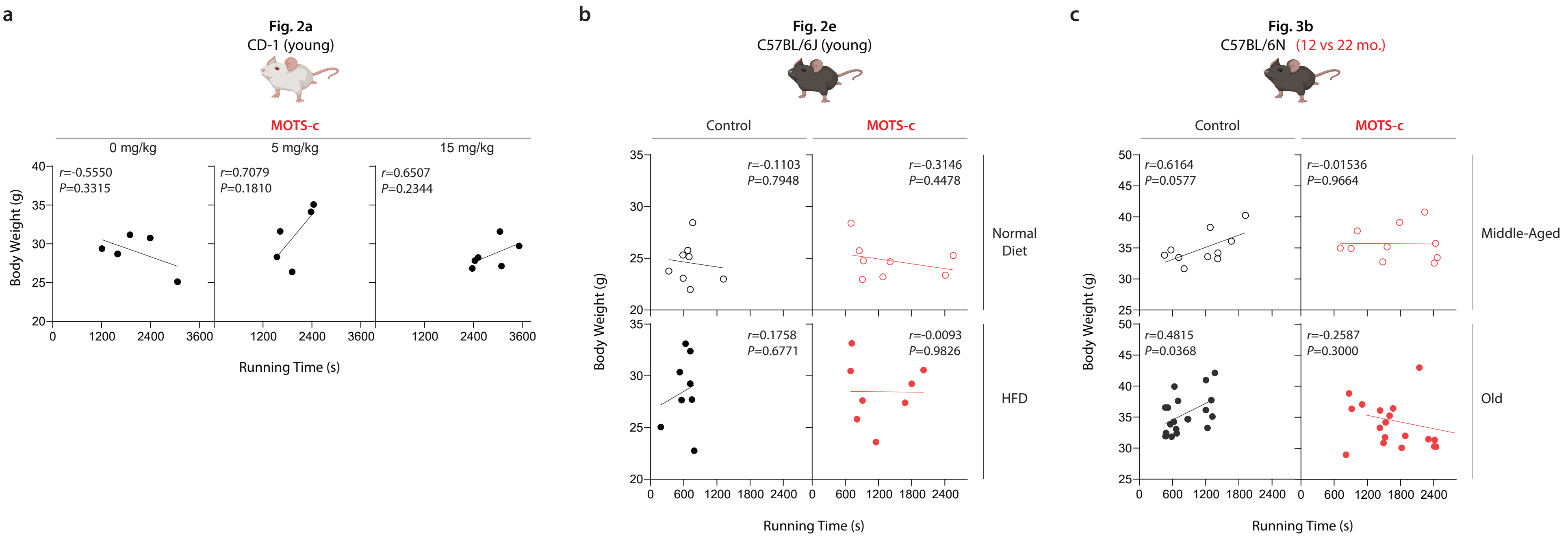

Supplementary Figure 7. Correlation between body weight and running capacity.
Correlation between body weight (g) and running time (s) was calculated based on Pearson correlation coefficient for **a** young CD-1 (see Fig. 2a), **b** young C57BL/6J (see Fig. 2e), and **c** older C57BL/6N (see Fig. 3b) mice.

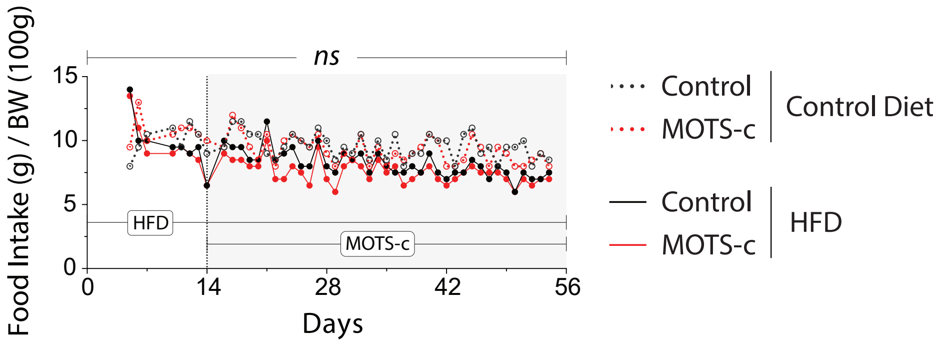

Supplementary Figure 8. The effect of MOTS-c on food intake in young C57BL/6J mice fed a normal or high-fat diet. Young C57BL/6J mice either on a HFD or a defined control diet were treated daily with MOTS-c (15 mg/kg/day; IP) or saline control (n=8) and food intake was measured. Mice were housed 4 animals/cage and food weight was measured per cage. Food intake is presented per mouse. Two-way ANOVA on 2 cages/group. The dotted line at Day 14 represents the start of MOTS-c treatment. Related to Fig. 2e-i.

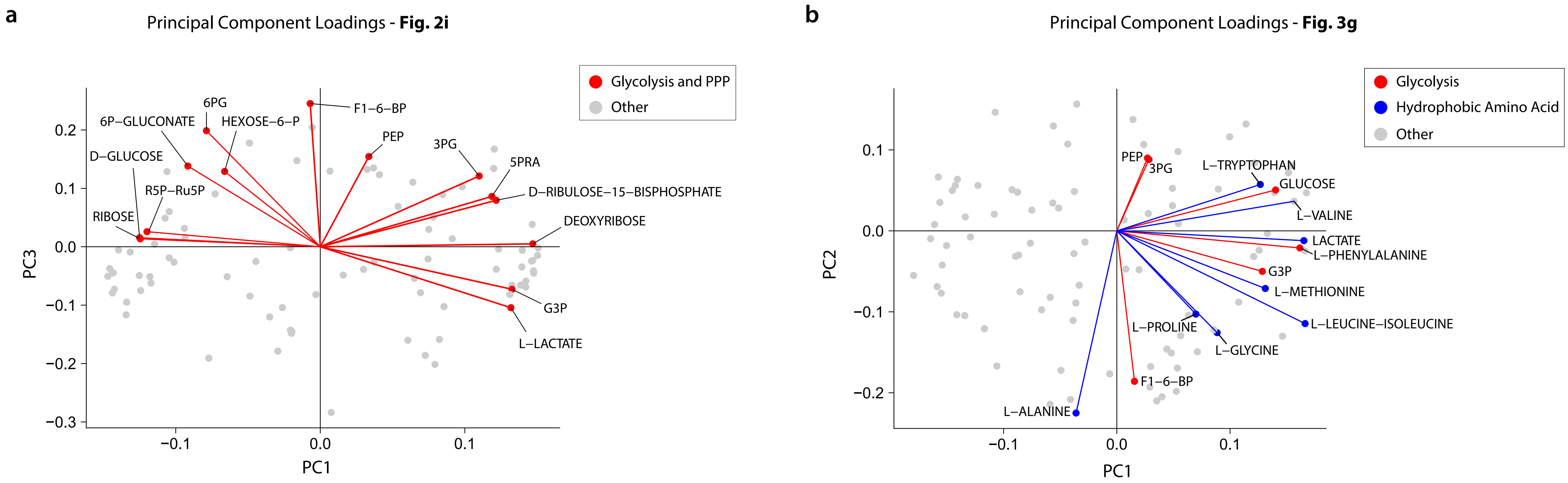

Supplementary Figure 9. Principal component loadings. PC loading **a** relevant to Fig. 2i, PC1 PC3 scatter plot with Glycolysis/ Pentose Phosphate Pathway labels and **b** relevant to Fig. 3g, PC1 PC2 scatter plot with Glycolysis and Hydrophobic Amino Acid labels.

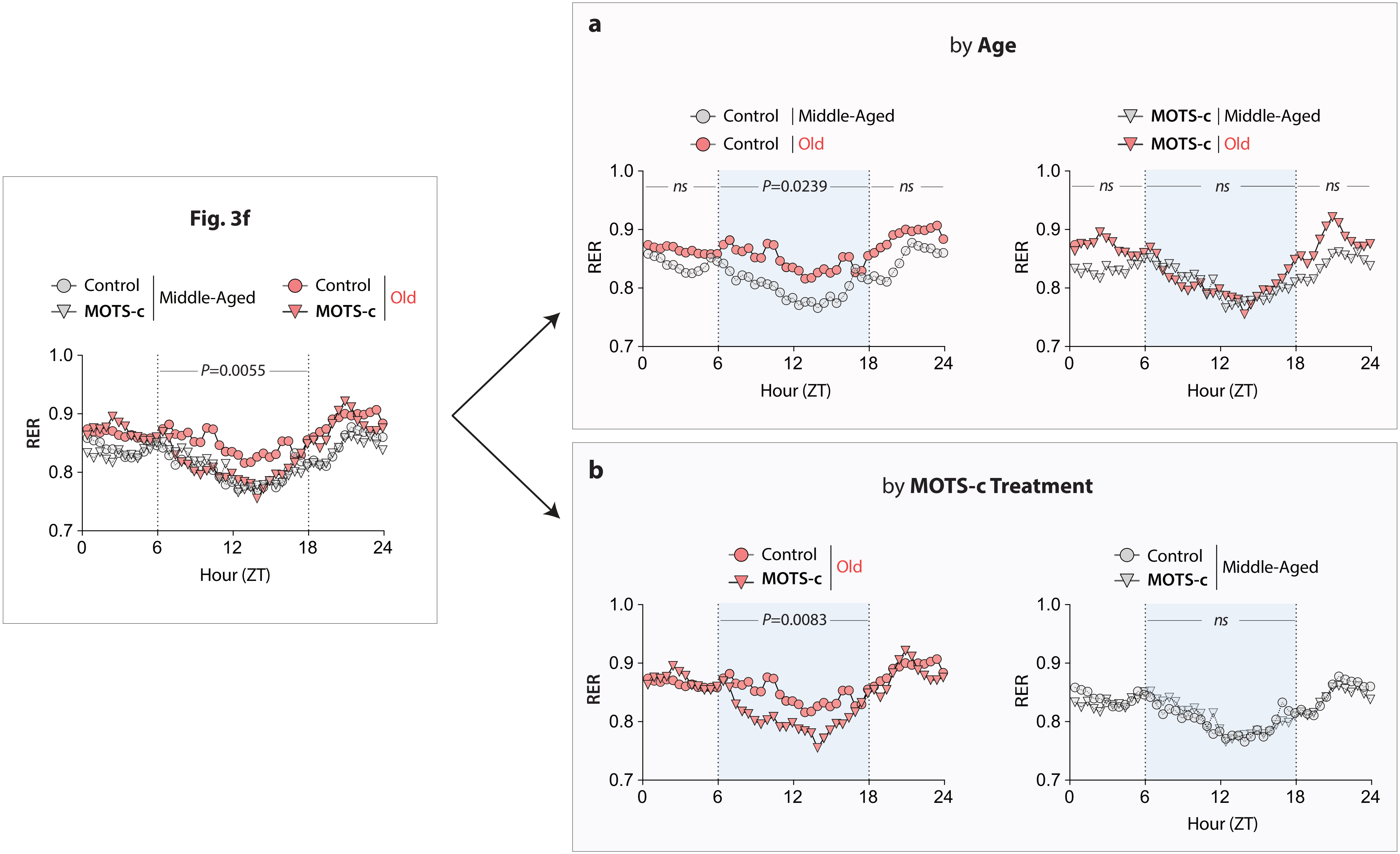

Supplementary Figure 10. Alternate presentation of Fig. 3f divided by age and MOTS-c treatment. P-values derived using two-way ANOVA. **a** P=0.0239. **b** P=0.0083.

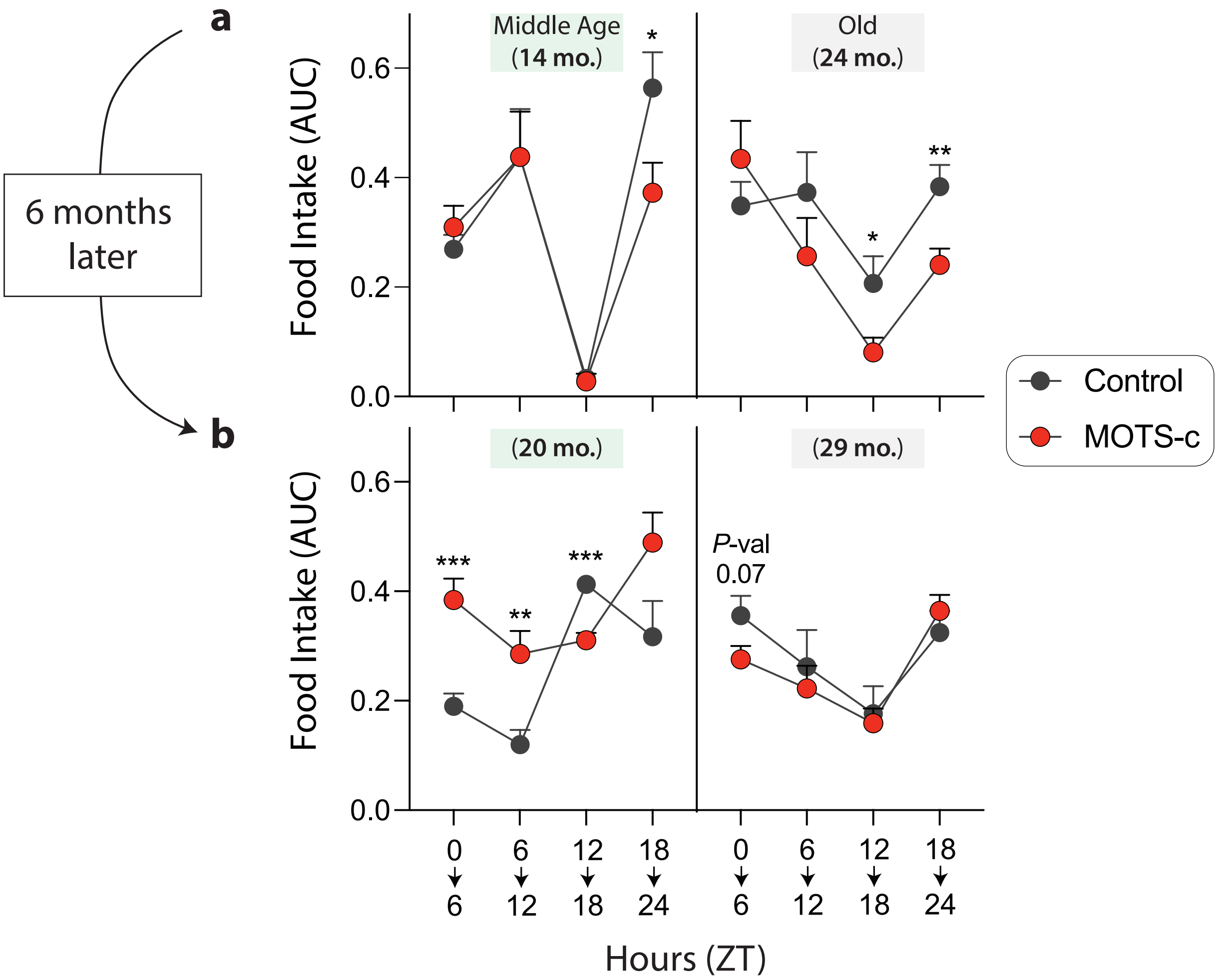

Supplementary Figure 11. Circadian pattern of food intake in MOTS-c treated old mice. a The sum of coninuous food intake measurements using metabolic cages divided into daily quartiles in MOTS-c treated middle-age (14 months) (*P*=0.0359) and old (24 months) (*P*=0.0370 and *P*=0.0089, respectively) mice (n=4). **b** Measurements were taken six months later in the same mice (*P*=0.0004, 0-6. *P*=0.0031, 6-12. *P*=0.000025, 12-18 and *P*=0.057 18-24). Data expressed as mean +/- SEM. Two-sided Student's t-test. **P*<0.05, ***P*<0.01, ****P*<0.001.

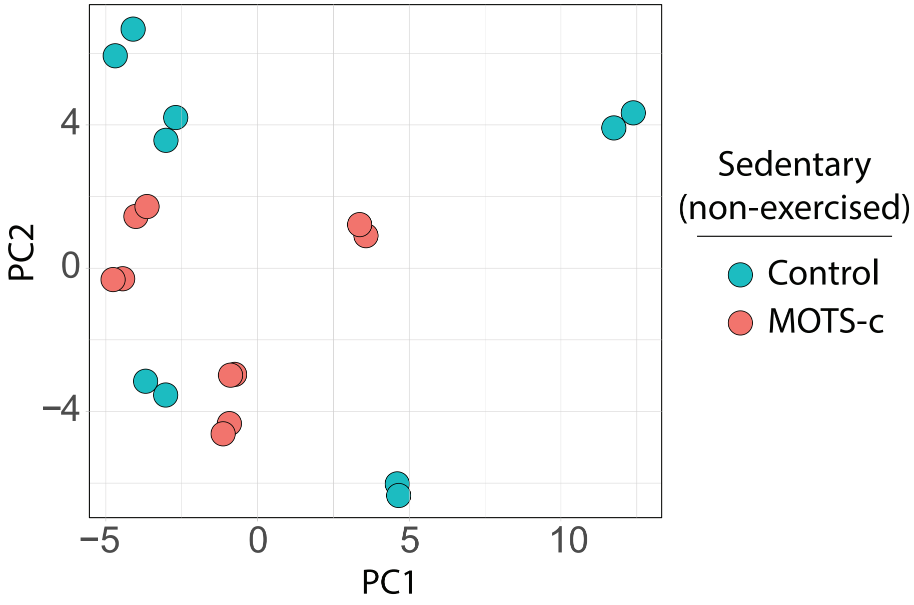

Supplementary Figure 12. Metabolomic analysis on sedentary MOTS-c-treated old mice. Skeletal muscle from sedentary (not treadmill-exercised) old mice (22.5 months) treated daily with MOTS-c (15 mg/kg/day) for 2 weeks (n=10) were subject to metabolomics and analyzed using PCA.

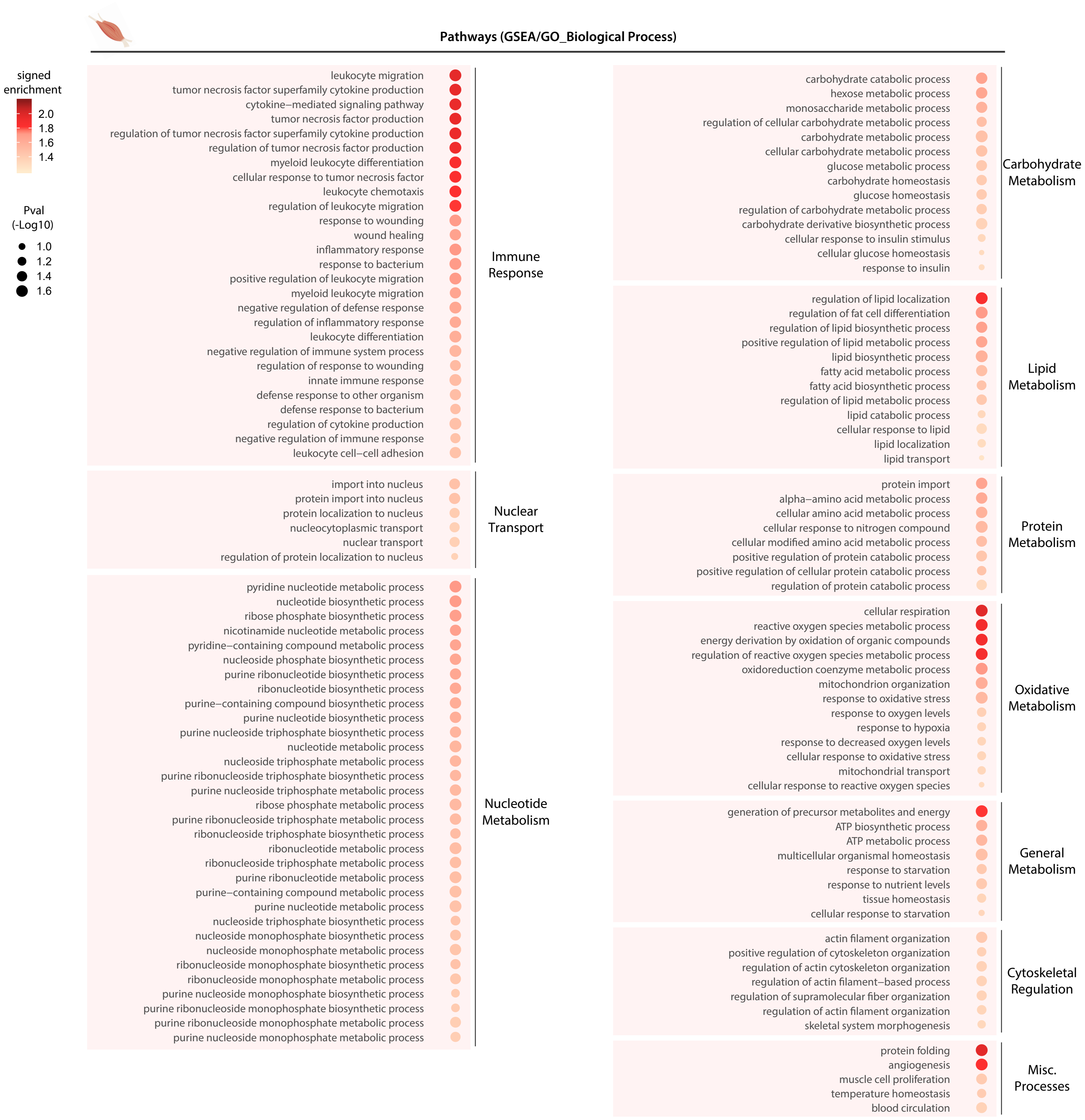

Supplementary Figure 13. Gene expression analysis on skeletal muscle from exercised MOTS-c-treated old mice. RNA-seq was performed on skeletal muscles from MOTS-c-treated old mice. Balloon plots of biological processes derived from Gene Set Enrichment Analysis (GSEA) using the Gene Ontology (Biological Process) database at a false discovery rate (FDR) < 15% (n=6).

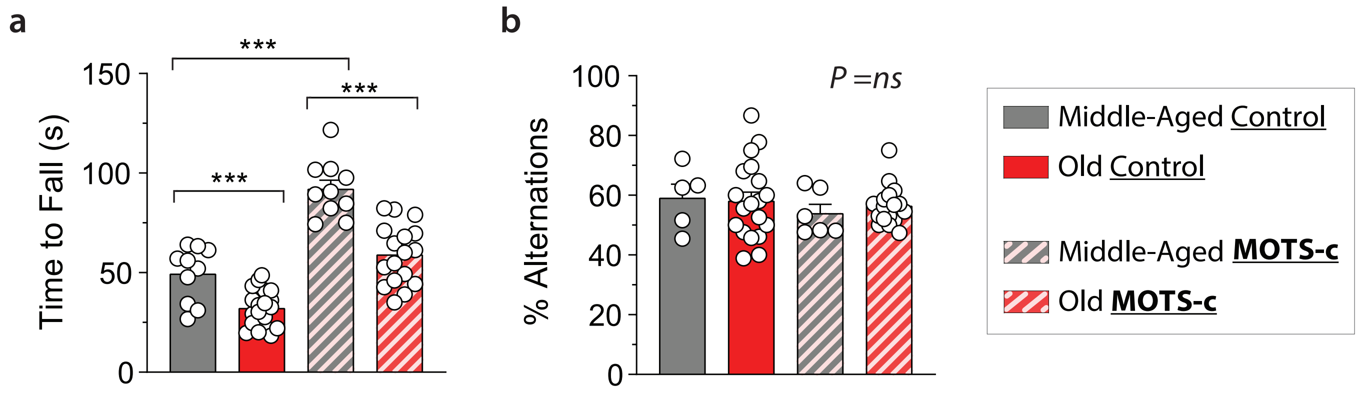

Supplementary Figure 14. Rotarod and Y-Maze tests in MOTS-c treated old mice. Middle-aged (14 mo.) and old (24 mo.) mice (n=10 middle-aged mice; n=17 old MOTS-c and n=19 old Control) were treated daily with MOTS-c (15 mg/kg/day;IP) and subject to **a** a rotarod test (*P*=0.000442, MA Control vs. Old Control, *P*=0.000008, MA MOTS-c vs. Old MOTS-c, *P*=0.000003, MA Control vs. MA MOTS-c, and *P*=0.0000002, Old Control vs. Old MOTS-c) and **b** y-maze test. Data expressed as mean +/- SEM. Two-sided Student's t-test. ****P*<0.0001.

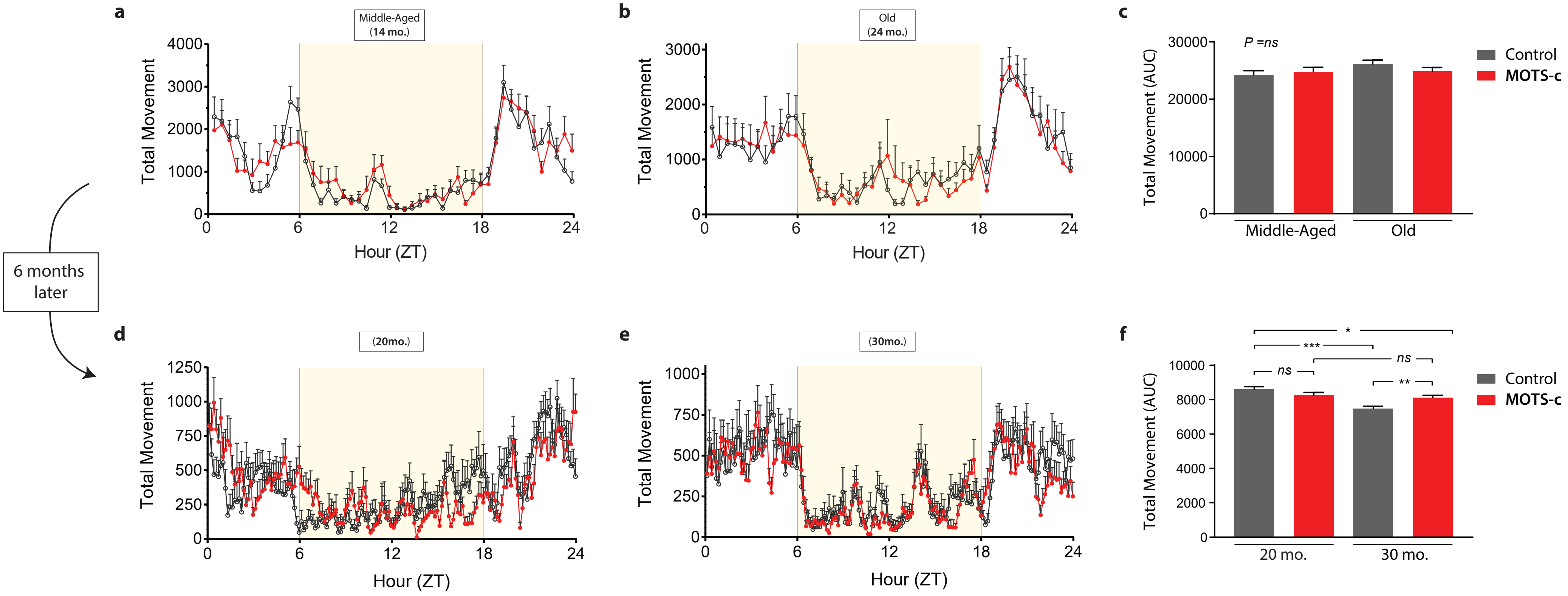

Supplementary Figure 15. Total Physical Activity in MOTS-c treated old mice. Total movement [horizontal and vertical movement (XYZ-axis)] of MOTS-c treated **a** middle-aged (14 mo.) and **b** old (24 mo.) mice were continuously measured using metabolic cages throughout the day for three days (n=4). **c** The sum of all measured movements is shown. **d-f** The procedure was repeated on the same mice after 6 months of LLII MOTS-c treatment (*P*=0.0029, Old Control vs. Old MOTS-c, *P*=0.026, Middle-Aged Control vs. Old MOTS-c, *P*=0.000013, Middle-Aged Control vs. Old Control. Data expressed as mean +/- SEM of three 24-hour acquisition cycles. Two-sided Student's t-test. **P*<0.05, ***P*<0.01, ****P*<0.0001.

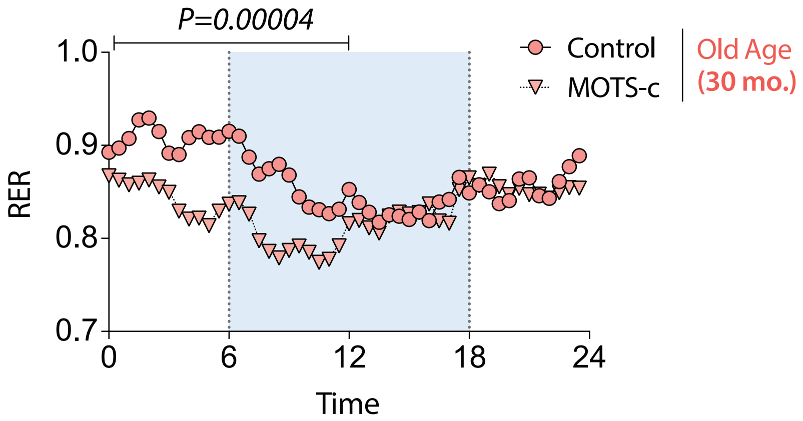

Supplementary Figure 16. MOTS-c-dependent circadian fuel selection old mice. Respiratory Exchange Ratio (RER) measurements in LLII MOTS-c-treated, or control, old mice (30 mo.; n=4). Shaded region represents daytime (light cycle) (P=0.00004). Data expressed as mean +/- SEM of three 24-hour acquisition cycles. Two-way ANOVA (repeated measures).

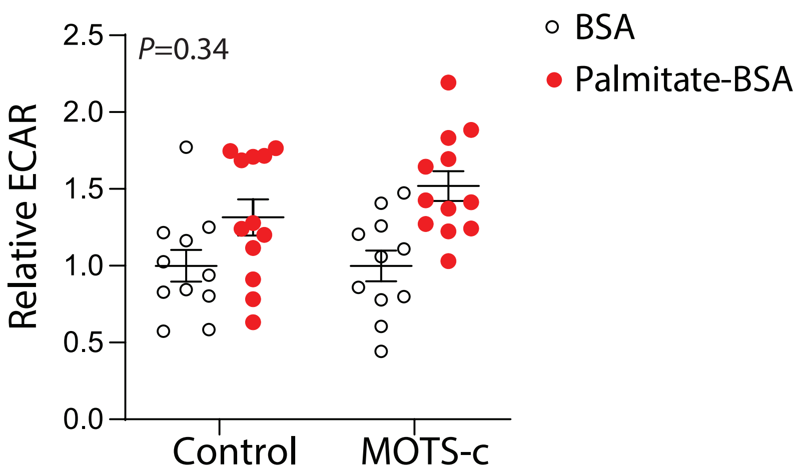

Supplementary Figure 17. MOTS-cdependent glycolytic rate in lipid-stimulated mouse myoblasts. C2C12 mouse myoblasts were treated with MOTS-c (10uM) or saline control in nutruent-limited media (n=11 BSA baseline, n=12 palmitate addition). Real-time glycolytic flux determined by the extracellular acidification rate was measured using the XF96 Seahorse bioanalyzer. Prior to the start of the assay, nutrient-deprived cells were given either BSA alone or palmitate bound to BSA (palmitate-BSA) to determine the capacity to metabolize fatty acids. Data expressed as mean +/- SEM. Two-Way ANOVA. *$P<0.05$, **$P<0.01$,***$P<0.001$.

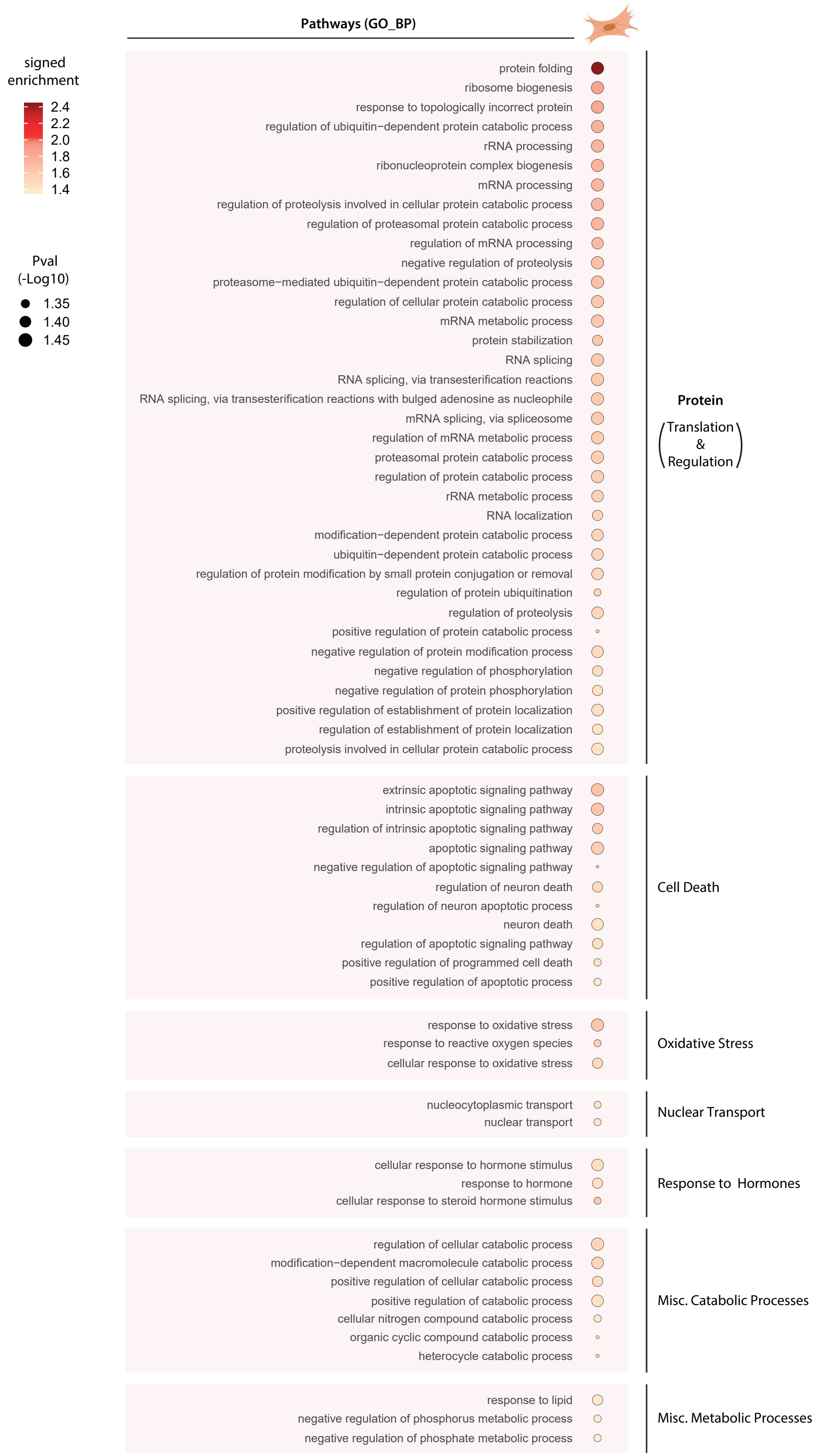

Supplementary Figure 18 Gene expression analysis on MOTS-c-treated mouse myoblasts under metabolic stress. RNA-seq was performed on C2C12 myoblasts following 48 hours of GR/SD with MOTS-c (10uM) treatment only once initially (n=6). Balloon plots of biological processes derived from Gene Set Enrichment Analysis (GSEA) using the Gene Ontology (Biological Process) database at a false discovery rate (FDR) < 15% (n=6).
