# Supplementary material for: MOTS-c is an exercise-induced mitochondrial-encoded regulator of age-dependent physical decline and muscle homeostasis
Source: Nat Commun. 2021 Jan 20;12:470. doi: 10.1038/s41467-020-20790-0 (PMC7817689; doi:10.1038/s41467-020-20790-0)
Supplement: Supplementary file 2 — Description of Additional Supplementary Files [file 41467_2020_20790_MOESM2_ESM.docx]

**Description of Additional Supplementary Files**

Supplementary Data 1: Young C57BL6J HFD Metabolomics Raw Data

Supplementary Data 2: Young C57BL6J HFD MSEA Results

Supplementary Data 3: Aged C57BL6N Metabolomics Raw Data

Supplementary Data 4: Aged C57BL6N MSEA Results

Supplementary Data 5: DESeq2 Analysis Results

Supplementary Data 6: Muscle Enrichment table GO BP FDR15 pathways significant in 0 or more

Supplementary Data 7: C2C12 Enrichment table GO-BP FDR5 pathways significant in 0 or more

Supplementary Data 8: Enrichment table GO BP FDR15 pathways significant in 2 or more

Supplementary Data 9: Muscle Enrichment table KEGG FDR15 pathways significant in 0 or more
