# Supplementary material for: MOTS-c is an exercise-induced mitochondrial-encoded regulator of age-dependent physical decline and muscle homeostasis
Source: Nat Commun. 2021 Jan 20;12:470. doi: 10.1038/s41467-020-20790-0 (PMC7817689; doi:10.1038/s41467-020-20790-0)
Supplement: Supplementary file 12 — Reporting Summary [file 41467_2020_20790_MOESM12_ESM.pdf]

nature research

Corresponding author(s): Changhan Lee
Last updated by author(s): Nov 17, 2020

Reporting Summary

Nature Research wishes to improve the reproducibility of the work that we publish. This form provides structure for consistency and transparency in reporting. For further information on Nature Research policies, see our Editorial Policies and the Editorial Policy Checklist.

Statistics

For all statistical analyses, confirm that the following items are present in the figure legend, table legend, main text, or Methods section.

n/a	Confirmed	
☐	☒	The exact sample size (*n*) for each experimental group/condition, given as a discrete number and unit of measurement
☐	☒	A statement on whether measurements were taken from distinct samples or whether the same sample was measured repeatedly
☐	☒	The statistical test(s) used AND whether they are one- or two-sided<br>*Only common tests should be described solely by name; describe more complex techniques in the Methods section.*
☐	☒	A description of all covariates tested
☐	☒	A description of any assumptions or corrections, such as tests of normality and adjustment for multiple comparisons
☐	☒	A full description of the statistical parameters including central tendency (e.g. means) or other basic estimates (e.g. regression coefficient) AND variation (e.g. standard deviation) or associated estimates of uncertainty (e.g. confidence intervals)
☐	☒	For null hypothesis testing, the test statistic (e.g. *F*, *t*, *r*) with confidence intervals, effect sizes, degrees of freedom and *P* value noted<br>*Give P values as exact values whenever suitable.*
☒	☐	For Bayesian analysis, information on the choice of priors and Markov chain Monte Carlo settings
☒	☐	For hierarchical and complex designs, identification of the appropriate level for tests and full reporting of outcomes
☐	☒	Estimates of effect sizes (e.g. Cohen's *d*, Pearson's *r*), indicating how they were calculated

Our web collection on statistics for biologists contains articles on many of the points above.

Software and code

Policy information about availability of computer code

Data collection	Principal component analysis (PCA) was performed using the mean-centered matrix of metabolite values per each mouse. Principal components that separated sample groups were identified with visual inspection. Loadings from principal components that stratify experimental samples versus controls were then queried against metabolic pathways using a Kolmogorov-Smirnov statistic against the expected distribution of metabolites. Metabolic pathway enrichment analysis (gene set enrichment analysis, GSEA) were performed using 28 metabolic pathways defined by the Kyoto Encyclopedia of Genes and Genomes (KEGG) database using pathways with four or more measured metabolites. PCA was performed using the R base package function 'prcomp' V4.0.3. The first 2 principal components were used. Statistical tests were performed using GraphPad Prism version 8. 1.2. Western blots were quantified using ImageJ version 1.51. DEseq2 normalized fold-changes were then used to estimate differential gene expression between control and MOTS-c treated muscle or cell samples using the 'DESeq2' R package (DESeq2 1.16.1) 78. The heatmap of expression across samples for significant genes was plotted using the R package 'pheatmap' 1.0.10. Putative protein-protein interaction was derived using the STRING (Search Tool for the Retrieval of Interacting Genes/ Proteins) database version 11.0.
Data analysis	The RNA-seq analytical code will be made available on the Benayoun lab github (https://github.com/BenayounLaboratory/MOTSc_Exercise).

For manuscripts utilizing custom algorithms or software that are central to the research but not yet described in published literature, software must be made available to editors and reviewers. We strongly encourage code deposition in a community repository (e.g. GitHub). See the Nature Research guidelines for submitting code & software for further information.

Data

Policy information about availability of data

All manuscripts must include a data availability statement. This statement should provide the following information, where applicable:

- Accession codes, unique identifiers, or web links for publicly available datasets
- A list of figures that have associated raw data
- A description of any restrictions on data availability

All data are available in the main manuscript and supplementary material. Additional data, including RNA-seq with PCA, KEGG and DESeq2 have been uploaded to the NCBI SRA database (accession: PRJNA556045).

Field-specific reporting

Please select the one below that is the best fit for your research. If you are not sure, read the appropriate sections before making your selection.

☒ Life sciences ☐ Behavioural & social sciences ☐ Ecological, evolutionary & environmental sciences

For a reference copy of the document with all sections, see nature.com/documents/nr-reporting-summary-flat.pdf

Life sciences study design

All studies must disclose on these points even when the disclosure is negative.

Sample size	The number of mice was determined based on (i) our experience and that of our collaborators, and (ii) sample size calculations to detect a 10-15% difference between groups with 80% power and significance level (alpha) of 0.05 (two-tailed). The survival data was not initially planned as a lifespan study, but time of death was recorded following the primary objective, which was physical capacity tests at old age. For cell culture studies, three biological replicates were analyzed in triplicate. Human studies were limited to ten participants as approved by the Northern Health and Disibility Ethics Committee (New Zealand).
Data exclusions	No data was excluded from analysis.
Replication	For cell culture experiments, biological triplicates were used and analyzed with technical triplicates as well. In mouse studies, experiments were replicated in various cohorts. Physical capacity experiments were done on each set of mice, but not repeated within cohorts (each mouse performed the test once) unless otherwise described.
Randomization	Random allocation
Blinding	Inbred and outbred mice were randomly distributed between control and experimental groups. One mouse operator was available for these experiments. Human muscle/plasma were subject to MOTS-c measurements and presented in its entirety as a single group - repeated measures for all participant entails that each person served as their own control.

Reporting for specific materials, systems and methods

We require information from authors about some types of materials, experimental systems and methods used in many studies. Here, indicate whether each material, system or method listed is relevant to your study. If you are not sure if a list item applies to your research, read the appropriate section before selecting a response.

Materials & experimental systems

n/a	Involved in the study
☐	☒ Antibodies
☐	☒ Eukaryotic cell lines
☒	☐ Palaeontology and archaeology
☐	☒ Animals and other organisms
☐	☒ Human research participants
☒	☐ Clinical data
☒	☐ Dual use research of concern

Methods

n/a	Involved in the study
☒	☐ ChIP-seq
☒	☐ Flow cytometry
☒	☐ MRI-based neuroimaging

Antibodies

Antibodies used	MOTS-c: 1:250 dilution, rabbit polyclonal; YenZym #YZ5828, USA<br>GAPDH: 1:1,000 dilution, cat# 5174; Cell Signaling, USA<br>Secondary HRP-conjugated antibodies: 1:30,000 dilution. (#7074; Cell Signaling, USA)
Validation	GAPDH: Validation Steps (Cell Signaling) Include

-Examination of several cell lines and/or tissues of known expression levels allows accurate determination of species cross-reactivity and verifies specificity.
-Treatment of cell lines with factors and conditions that alters the expression of target protein/peptide.
-Phosphatase treatment confirms phospho-specificity.
-The use of siRNA transfection or knockout cell lines verifies target specificity.
-Side-by-side comparison of lots to ensures lot-to-lot consistency.
-Optimal dilutions and buffers are predetermined, positive and negative cell extracts are specified, and detailed protocols are already optimized, saving valuable time and reagents.

MOTS-c: Validated through YenZym, and through various recent publications confirming the specificity of the MOTS-c antibody.

Eukaryotic cell lines

Policy information about cell lines

Cell line source(s)	C2C12 cells from ATCC
Authentication	C2C12 cells were authenticated through ATCC per certificate. Additionally, routine morphology checks and growth curve analysis were performed.
Mycoplasma contamination	Negative per ATCC certificate.
Commonly misidentified lines (See ICLAC register)	No commonly misidentified cell lines were used in this study.

Animals and other organisms

Policy information about studies involving animals; ARRIVE guidelines recommended for reporting animal research

Laboratory animals	12-week old male CD-1 (outbred) mice (Charles River, USA), 12-week old male C57BL/6J mice (Jackson Laboratory) and 8- and 18-month old male C57BL/6N mice (National Institute on Aging; NIA) were obtained. NIA mice were sufficiently acclimated for 4 months in our vivarium until they were considered middle-aged (12 mo.) and old (22 mo.). mice were housed 3-4 per cage in a facility with a 12:12 hour light- dark cycle (light period 0600-1800) at 24ºC. Food and water were available ad libitum.
Wild animals	No wild animals were used in this study.
Field-collected samples	No field-collected samples were used in this study.
Ethics oversight	All animal work was performed in accordance with the University of Southern California (USC) Institutional Animal Care and Use Committee.

Note that full information on the approval of the study protocol must also be provided in the manuscript.

Human research participants

Policy information about studies involving human research participants

Population characteristics	10 sedentary (<4h aerobic exercise/week) healthy young males (24.5 ± 3.7 years old and BMI 24.1 ± 2.1) were recruited to take part in a two-visit exercise trial. Recruited participants were free of cardiovascular, metabolic and blood diseases and were not taking any medication or supplements.
Recruitment	Participants were recruited via local advertisements (posters and emailing lists) at the University of Auckland.
Ethics oversight	Participants gave written consent before the commencement of the study, which was approved by the Northern Health and Disability Ethics Committee (New Zealand) (16/STH/116/AM0l).

Note that full information on the approval of the study protocol must also be provided in the manuscript.
